# Meet the Editorial Board Member

**DOI:** 10.2174/1570159X2205231107094531

**Published:** 2023-11-07

**Authors:** Marco Fiore

**Affiliations:** 1 Institute of Biochemistry and Cell Biology, IBBC-CNR, Rome, Italy

   Researcher at the Institute of Biochemistry and Cell Biology of the Italian National Research Council IBBC-CNR. Graduated in Natural Sciences and in Biological Sciences at the Sapienza University of Rome, Italy. PhD in Medical Sciences at the Psychiatric Department of the University of Groningen, The Netherlands. He potentiated his professional experience also at the Institute of Zoology, Oxford University, UK and at the Department of Physiology and Behavior, University of California at Davis, CA-USA.

## References

[1] Terracina S., Ferraguti G., Tarani L., Messina M.P., Lucarelli M., Vitali M., De Persis S., Greco A., Minni A., Polimeni A., Ceccanti M., Petrella C., Fiore M. (2022). Transgenerational abnormalities induced by paternal preconceptual alcohol drinking. findings from humans and animal models.. Curr. Neuropharmacol..

[2] Fiore M., Tarani L., Radicioni A., Spaziani M., Ferraguti G., Putotto C., Gabanella F., Maftei D., Lattanzi R., Minni A., Tarani F., Petrella C. (2021). Serum prokineticin-2 in prepubertal and adult klinefelter individuals.. Can. J. Physiol. Pharmacol..

[3] Meschino N., Laviola G., Busdraghi L.M., Petrella C., Fiore M. (2021). Aberrant early in life stimulation of the stress-response system affects emotional contagion and oxytocin regulation in adult male mice.. Int. J. Mol. Sci..

[4] Petrella C., Di Certo M.G., Gabanella F., Barbato C., Ceci F.M., Greco A., Ralli M., Polimeni A., Angeloni A., Severini C., Vitali M., Ferraguti G., Ceccanti M., Lucarelli M., Severi C., Fiore M. (2021). Mediterranean diet, brain and muscle: olive polyphenols and resveratrol protection in neurodegenerative and neuromuscular disorders.. Curr. Med. Chem..

[5] Laviola G., Leonardo A., Ceci F.M., Fiore M. (2021). Callous unemotional trait-like mice and their stressed dams.. Psychoneuroendocrinology.

[6] Ceci F.M., Ferraguti G., Petrella C., Greco A., Tirassa P., Iannitelli A., Ralli M., Vitali M., Ceccanti M., Chaldakov G.N., Fiore M. (2021). NGF, stress and diseases.. Curr. Med. Chem..

[7] Petrella C., Ferraguti G., Tarani L., Chaldakov G.N., Ceccanti M., Greco A., Ralli M., Fiore M. (2021). Olive polyphenols and chronic alcohol protection.. olives and olive oil in health and disease prevention 2nd edition..

[8] Ciafrè S., Ferraguti G., Greco A., Polimeni A., Ralli M., Ceci F.M., Ceccanti M., Fiore M. (2020). Alcohol an early life stressor: epigenetics, metabolic, neuroendocrine and neurobehavioral implications.. Neurosci. Biobehav. Rev..

[9] Marisa P.M., Maria G.P., Carla P., Mario V., Antonio G., Massimo R., Mauro C., Giampiero F., Isabella N., Alba R., Marco F. (2021). Alessio, D’Angelo. Advanced midwifery practice: intrapartum ultrasonography to assess fetal head station and comparison with vaginal digital examination.. Minerva Obstet. Gynecol..

[10] Ferraguti G., Terracina S., Micangeli G., Lucarelli M., Tarani L., Ceccanti M., Spaziani M., D’Orazi V., Petrella C., Fiore M. (2023). NGF and BDNF in pediatrics syndromes.. Neurosci. Biobehav. Rev..

